# Selection scan reveals three new loci related to high altitude adaptation in Native Andeans

**DOI:** 10.1038/s41598-018-31100-6

**Published:** 2018-08-24

**Authors:** Vanessa C. Jacovas, Cainã M. Couto-Silva, Kelly Nunes, Renan B. Lemes, Marcelo Z. de Oliveira, Francisco M. Salzano, Maria Cátira Bortolini, Tábita Hünemeier

**Affiliations:** 1 Genetics Departament, Biosciences Institute, Federal University of Rio Grande do Sul, Porto Alegre, RS Brazil; 2 Department of Genetics and Evolutionary Biology, Biosciences Institute, University of São Paulo, São Paulo, SP Brazil; 3 Vale do Rio dos Sinos University, São Leopoldo, RS Brazil

## Abstract

The Andean Altiplano has been occupied continuously since the late Pleistocene, ~12,000 years ago, which places the Andean natives as one of the most ancient populations living at high altitudes. In the present study, we analyzed genomic data from Native Americans living a long-time at Andean high altitude and at Amazonia and Mesoamerica lowland areas. We have identified three new candidate genes - *SP100*, *DUOX2* and *CLC* - with evidence of positive selection for altitude adaptation in Andeans. These genes are involved in the *TP53* pathway and are related to physiological routes important for high-altitude hypoxia response, such as those linked to increased angiogenesis, skeletal muscle adaptations, and immune functions at the fetus-maternal interface. Our results, combined with other studies, showed that Andeans have adapted to the Altiplano in different ways and using distinct molecular strategies as compared to those of other natives living at high altitudes.

## Introduction

Along their great expansion, humans have inhabited almost all environments in the five continents. Among several harsh environments that were occupied, the highlands are probably the ones that needed more adaptations for survival^1^. At least in three geographically distinct locations have this evolutionary adaptation been studied: Andean Altiplano (South America), Himalaya (China/Tibet, Asia) and Semien Mountain (northern Ethiopia, Africa) Plateaus. Andes have been peopled continuously since the late Pleistocene, ~12,000 yBP^2^ while the time of settlement and permanent occupation of both Tibet and Ethiopia remain a topic of debate, varying widely^3,4^. Despite some uncertainties in the permanent occupation dating, it is certain that humans have inhabited these regions of hostile climates for thousands of years.

Several physiologic factors are associated with living at high altitude (≥2,500 meters where only 75% of the oxygen available at sea level occurs; http://www.altitude.org/air_pressure.php), including adaptations for high ultraviolet radiation index, thermal amplitude, and changes in the pulmonary capacity due to hypoxia^5,6^. High altitude leads to a rapid physiologic/adaptive response in individuals from lowlands; however, prolonged exposure to environmental-related factors might have harmful outcomes. Remarkable features such as increased pulmonary function, hypoxia tolerance, and increased hemoglobin levels have been observed in Andean populations^7^. How such adaptations took place is still not clear, and just a few genes have been associated with the high altitude adaptation phenotype in human populations^8–13^.

Interestingly, the set of genes presenting signs of natural selection changes according to high altitude, indicating that under an analogous selective pressure, different genetic solutions have emerged. For instance, genomic scans for selection have revealed at least 40 candidate genes related to the Hypoxia Inducible Factor (HIF), such as *EPAS1* in populations from Tibet, *EGLN1* in Andeans and Tibetans and *THRB* and *ARNT2* in Ethiopians^8,14–18^. The populations from the Andean plateau also presented signs of natural selection in other genes, such as *BRINP3*, *NOS2*, and *TBX5*, involved in the nitric oxide pathway (NOS) and related to cardiovascular health^12^. In addition, Jacovas *et al*.^19^ using the candidate gene approach inferred that a combination of some derived and ancestral alleles of *USP7, LIF* and *MDM2* genes, all three in the *TP53* pathway, could have been essential for the successful establishment of Native American populations in the Andean highlands.

Since different investigations pointed to distinct sets of genes involved in high altitude adaptation, more studies are necessary to fully understand the different genetic landscapes present in highland populations around de world. In the present study, we compared genomic data from Native American populations living for a long-time at high altitude (Andean Altiplano) with those living at lowlands (Amazon and Mesoamerica), with the purpose of expanding our knowledge about the genetic repertoire responsible for the successful human colonization of the Andes.

## Results

### Natural selection analysis

**Figure 1:**
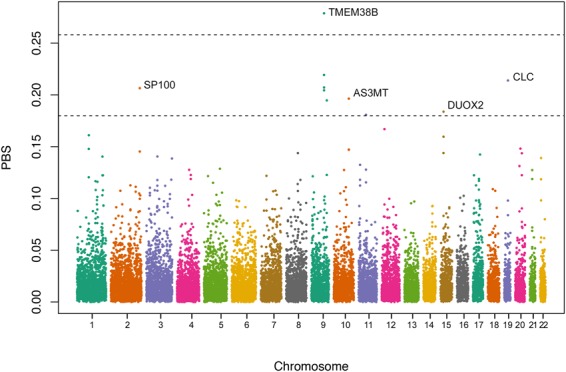
Average PBS values in windows of 20 SNPs, using a step size of 5 SNPs. The 99.5th and 99.9th percentiles of the empirical distribution are shown as black dashed horizontal lines. Names of genes associated with the highest peaks are shown.

Population Branch Statistic (PBS) values were estimated for each individual SNP. To avoid spurious results due to single SNPs, windows of 20 SNPs were used to estimate the mean PBS values for a given region. Then, we checked the outliers’ peaks, above the 99.5th and 99.9th percentiles, to identify in each outlier window the SNPs with the highest PBS value and assigned the gene to which it belonged (or the nearest gene). Based on this approach, five candidate genes were identified: *SP100* (SP100 Nuclear Antigen), *TMEM38B* (Transmembrane Protein 38B)*, AS3MT* (Arsenite 3 Methyltransferase), *DUOX2* (Dual Oxidase 2) and *CLC* (Charcot-Leyden Crystal Galectin, also known as Galectin-10) (Table 1 and Fig. 1). Among these candidate genes, *AS3MT and TMEM38B* have been identified in previous scans for natural selection in Andeans^13,20^.

**Table 1:** Population Branch Statistic (PBS) individual values and Cross-Population Extended Haplotype Homozygosity (XP-EHH) for all SNPs found under selection in Native Andean populations.

SNP	Alelle		Gene	Position	PBS	XPEHH			
	Ancestral	Derived				Andean *vs*. Mesoamerican	*p*-value	Andean *vs*. Amazonian	*p*-value
rs13411586	C*	T	*SP100*	230988046	0.5846	2.3789	0.0037	2.1703	0.0065
rs9678342	C*	T	*SP100*	230991955	0.5547	2.3193	0.0044	2.1356	0.0071
rs7582700	T*	C	*SP100*	231024349	0.4644	2.2704	0.0050	2.1074	0.0076
rs7039618	A	G*	*TMEM38B*	107497627	0.3618	0.0842	0.3312	0.5672	0.1458
rs3817141	T*	C	*TMEM38B*	107507950	0.3906	0.0255	0.3099	0.6205	0.1351
rs10978213	G*	A	*TMEM38B*	107511706	0.3618	0.0235	0.3092	0.6171	0.1358
rs10816302	A*	G	*TMEM38B*	107526354	0.3835	0.0937	0.2697	0.6664	0.1264
rs10978240	A	G*	*TMEM38B*	107575093	0.3923	0.0764	0.2753	0.6307	0.1331
rs1046778	T	C*	*AS3MT*	104651474	0.3124	0.5023	0.5118	0.4008	0.4631
rs269866	G*	A	*DUOX2*	43181698	0.6185	2.0599	0.0086	2.5865	0.0021
rs440191	A	G*	*CLC*	44913483	0.3039	1.6207	0.0234	0.3166	0.2046

Ancestral and derived alleles according to the 1000 Genomes data.

^*^Putative selected alleles.

**Figure 2:**
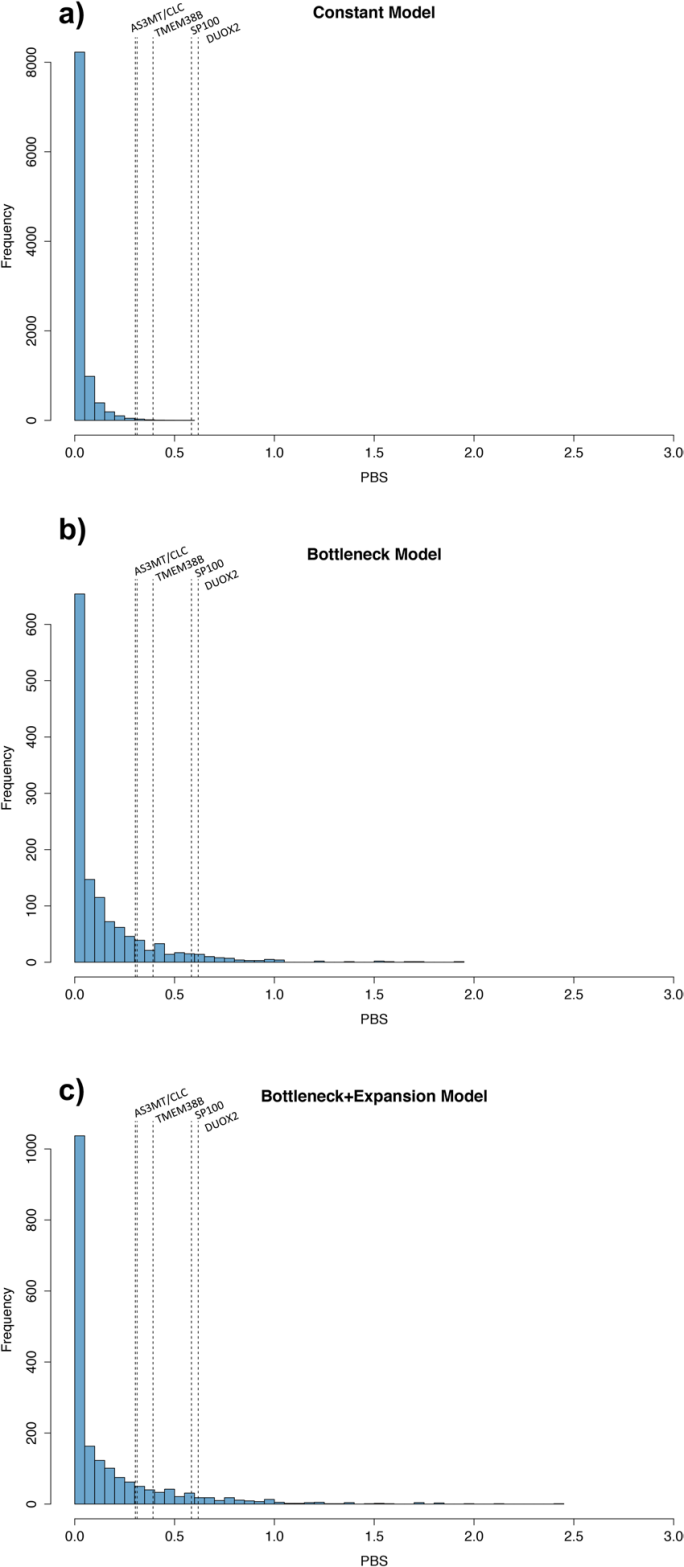
Distribution of 10,000 simulated PBS values under three neutral coalescent models. (**a**) Constant population model. (**b**) Population bottleneck model; and (**c**) Population bottleneck followed by expansion model. The dashed line represents the top observed PBS SNP values in the empirical datasets.

Neutral coalescent simulations indicated that these deviations were statistically significant (p-values ranging between 0.03 and 0.0001; Fig. 2, Table S1), consistent with the action of positive selection as opposed to genetic drift in increasing the frequency of the putative selected alleles at all five tested loci. In addition, we applied the Cross-Population Extended Haplotype Homozygosity (XP-EHH) test to the same regions. The XP-EHH results also show significant differences between the Andean and Mesoamerican groups in three SNPs (rs13411586, rs9678342, rs7582700) of *SP100* and one SNP (rs269866) of *DUOX2* (Table 1). These SNPs, which are under putative selection in the PBS analysis with the most extreme values (0.46 to 0.62), also present significant XP-EHH values ≥ 2 in both Andean vs Mesoamerican and Andean vs Amazonian groups.

The observed allele density provided by the iHS test showed a notable Gaussian distribution pattern for all three groups (Fig. S1), with homozygosity decaying according to the distance from the focal markers.

**Figure 3:**
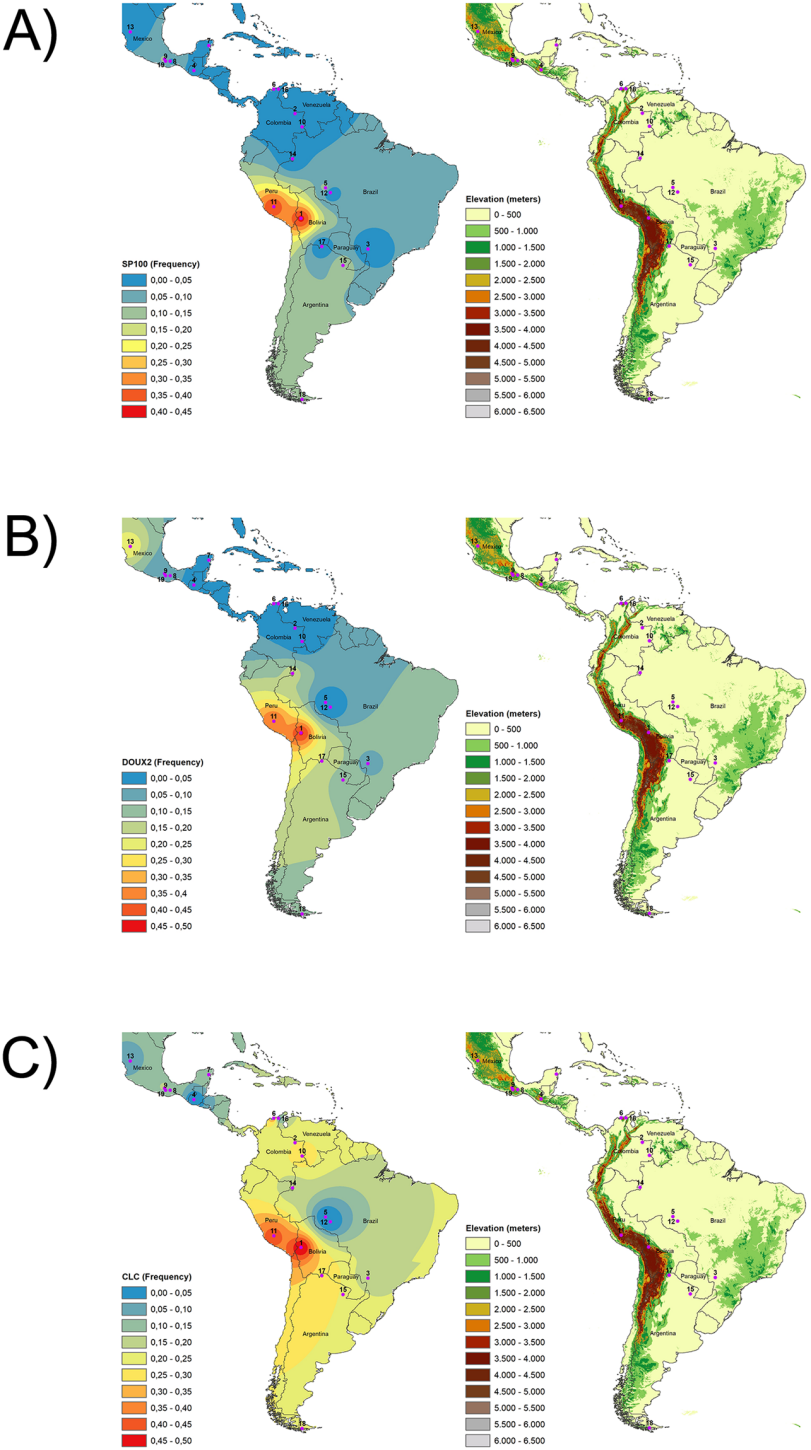
(**a**) rs13411586_C (*SP100*). (**b**) rs269866_G (*DUOX2*) and (**c**) rs440191_A (*CLC*) allele frequency distributions according to altitude. Populations (n ≥ 3): 1. Aymara, 2. Guahibo, 3. Guarani, 4. Kaqchikel, 5. Karitiana, 6. Kogi, 7. Maya, 8. Mixe, 9. Mixtec, 10. Piapoco, 11. Quechua, 12. Surui,13. Tepehuano, 14. Ticuna, 15. Toba, 16. Wayuu, 17. Wichi, 18. Yaghan and 19. Zapotec.

It should be noted that the distribution of alleles C (rs13411586, *SP100*), G (rs269866, *DUOX2*) and G (rs440191, *CLC*), which presented the highest PBS values (Table 1), showed their highest values in areas of very high Andean altitudes (Table 2 and Fig. 3).

**Table 2:** Frequencies of the putatively selected alleles in the populational groups.

Population (n)	*DUOX2* G allele (rs269866)	*SP100* C allele (rs13411586)	*CLC* G allele (rs440191)
**Mesoamerican Lowland (<2,500 m.)**			
Total (153)	0.068*	0.045*	0.128*
**South American (Andean) Highland (≥4,000 m.)**			
Total (63)	0.420*	0.397*	0.452*
**South American (Amazonian) Lowland (<2,500 m.)**			
Total (106)	0.048*	0.053*	0.142*

^*^Weighted average.

Bootstrap simulations indicated that in all instances the 95% confidence interval of allele frequencies in lowlanders does not include the average values observed for populations living in high altitudes (>4000 m above sea level) (Fig. S2), suggesting that the differences found in allele frequencies between population groups might be caused by a non-random evolutionary process.

### Effects of putatively selected alleles on gene expression

Homozygotes for the *DUOX2* putatively selected allele (rs269866 G) presented a slight increase in the expression of the DUOX2 protein (Fig. S3). Multiple testing across tissues showed significant expression of this protein in thyroid (m-value = 1.0), lungs (m-value = 0.996) and aorta artery (0.996) (Fig. S4). Homozygotes for the rs13411586 (*SP100*) putatively selected allele (C) presented an increase in the expression of the SP100 protein in skeletal muscles (Fig. S5). Multiple testing across tissues showed significant expression of this protein in skeletal muscle (m-value = 1.0) and testis (m-value = 0.971) (Fig. S6). There is no information available about the *CLC* gene expression profile.

## Discussion

We identified five loci under positive selection in Andean Native populations. Two of them were previously described: *AS3MT* was found to be under positive selection in Colla Andeans systematically exposed to arsenic water^20^ while *TMEM38B* reduced the negative effects of polycythemia (elevated hematocrit or decreased plasma volume) at high altitudes^13^. Three other genes, *SP100*, *DUOX2*, and *CLC* were identified for the first time in a high-altitude context in the present study. These genes are part of the *TP53* pathway, already indicated as a potential candidate to be under natural selection in high altitude populations^19,21^.

*SP100* is a single-copy gene in the human genome that produces several alternatively spliced Sp100 protein isoforms known as modulators of the p53 activity^22^. We found three SNPs in the *SP100* gene with high and significant PBS values, as well as significant XP-EHH values when Andeans were compared to others. One of these SNPs, rs13411586, is differentially expressed in humans; our prediction showed that individuals homozygous for the putatively selected allele (C) have increased Sp100 production.

Interestingly, we also identified that the *SP100* gene is differentially expressed in skeletal muscles (Fig. S3). Studies have revealed that a member of the HIF pathway, HIF-1, plays an important role in the regulation of oxygen homeostasis, which includes the physiological skeletal and heart muscle adaptations in situations of oxygen reduction due to muscular effort^23–25^ and ischemic cardiomyopathy, respectively^26^. Exposure to high altitude leads to reduced muscle mass and performance (*e.g*. lower work capacity and standing fatigue), except when one is evolutionarily adapted to it^27–29^.

HIF-1 protects cell-survival during low oxygen supply, while p53 promotes genome cell-death under hypoxia. The reason for these apparently antagonistic roles can be in the difference of the oxygen quantity available; in a normal condition, both p53 and HIF-1 levels are low, but in mild hypoxia, the p53 level remains low, whereas the HIF-1 level increases, protecting cells still relatively healthy from destruction. In severe hypoxia, p53 accumulation promotes the repression or degradation of anti-apoptotic proteins like HIF-1, inducing apoptosis of the damaged cells^30–32^. Sp100 is known as a modulator of the p53 activity^22^ and under tissue hypoxia due to ischemia, it is downregulated, leading to genomic instability^26^. The Andean population presents high allele C (rs13411586) frequency (Table 2), which in homozygosis increase Sp100 production according to our prediction test. Our result suggests an evolutionary solution to keep Sp100 at an adequate level in an environment with a constant low oxygen level. Furthermore, it is possible to speculate that there is an intricate balance in the level of expression of the *SP100, TP53* and *HIF-1* genes under hypoxia, considering both short (reversible physiological and metabolic adaptations) and long-term evolutionary adaptation scenarios.

DUOX2, expressed in epithelial cells of various tissues including nasal and lung, participates in the hydrogen peroxide (H_2_O_2_) pathway, which is required in the final steps of thyroid hormones production. It is also involved in Reactive Oxygen Species (ROS), a byproduct of the normal oxygen metabolism even under normal physiologic conditions^33^. However, different stressor conditions can increase the ROS production, *i.e*. high-altitude exposure (hypoxia and UV exposure), and pathological conditions such as cancer^34^. Salmeen *et al*.^35^ provided evidence that DUOX2 plays a role in a p53-dependent checkpoint mechanism for cell cycle entry.

*In vitro* and *in vivo* experiments showed that oxidative stress and generation of ROS caused by DUOX2 overexpression, in both hypoxia and hyperoxia, contribute to inflammation, carcinogenesis and cell death^36–42^. For instance, a functional study^36^ showed that under hyperoxia conditions, mutant mice for DUOX2 had significant lower acute lung injuries induced by hyperoxia. This finding pointed to the importance of these proteins in the response to changes of oxygen concentration in the environment. Another study^37^ found that chickens submitted to hypoxia (>3,000 m) had increased activity of DUOX/NOX proteins, indicating the physiological role of these enzymes in the process of adaptation to oxidative stress.

Our results on the expression of the *DUOX2* putatively selected allele G (high PBS values and significant XP-EHH value > 2; Table 1) also pointed to higher levels of protein expression in humans, mainly in the lungs and arteries. It is noteworthy that ROS contributes to inflammation in the vessel walls. Kim & Byzowa^43^ demonstrated that ROS has an important role in angiogenesis, a process of new blood vessel growth. Angiogenesis is a key event in the physiological response to hypoxia and therefore might have a role in the adaptation to high altitude in long-term residents, especially in individuals with excessive erythropoiesis (like those found in the Chronic Mountain Sickness [CMS] phenotype), to compensate a plausible change in microcirculation^44,45^.

SNP rs440191 is located at the 3′UTR region of *CLC*, and the putatively selected allele G is in complete linkage disequilibrium with the *CLC* rs395892 G allele in the Mexican population^46^. The latter is associated with eosinophil and basophil counts^47^, while rs440191 has so far been investigated just in approaches assessing allergic susceptibilities^48^. Gene expression queries did not show any significant eQTL related to this polymorphism, preventing any prediction of tissue-specific expression.

*CLC* (galectin-10) is still a poorly studied gene when compared to other members of the functionally polyvalent galectin family. It is recognized as a lysophospholipase expressed in eosinophils and basophils, although some authors identified it just as an enzyme that interacts with lysophospholipases^49^. The only functional study regarding this protein showed that hypoxia increases eosinophil accumulation and CLC production in humans, concomitant with a delay in constitutive apoptosis, antagonizing the normal pro-apoptotic effect of agents that normally induce eosinophil apoptosis^50^.

Regulation by the p53 transcription factor seems to be important in the galectin family genes’ expression. For instance, the galectin-3 gene has a binding site for p53, and p53 increases the transcription of paralogue galectin-7^51–53^. Altered expression of galectin genes, including *CLC*, was implicated in cancer emergence and progression, highlighting the role of the galectins in cell proliferation via cell death programs^54^.

Investigations with galectin paralogues have shown that galectin-1 in the first term ovine gestation placenta prevented inflammatory processes that harm the fetus^55^, while galectin-13, which has the highest homology to CLC, is a member of the group of the so-called “pregnancy-related proteins”, due to its special immune functions at the feto-maternal interface^56,57^. These fundamental cell functions, already described for humans and other placental mammals, may indicate the path that connects our CLC findings and the selection pressure in the Andean hostile climate.

In conclusion, our results pointed to a complex adaptation that occurred in Andean natives, which involved the *CLC, SP100* and *DUOX2* genes, not previously correlated in contexts of long-time adaptation to high altitudes. We also reinforced the role of the *TP53* pathway at least for the adaptation to the Andean environmental stresses. Combined with other studies, and incorporating the present one, it is clear that Andeans have adapted to the Altiplano in different ways and using distinct molecular strategies than those of other natives living at high altitude.

## Methods

### Populations

We analyzed 213,987 SNPs determined with Illumina 610quad from 63 Native Americans living at extreme high altitude (≥4,000 m; 63% of the oxygen available at sea level; http://www.altitude.org/air_pressure.php) and 259 living at lowland areas (<2,500 m), data previously published by Reich *et al*.^58^. Highlanders included Aymara and Quechua Andeans, while lowlanders were represented by 25 populations from the Mesoamerican and South American lowlands. Details about these populations, sample sizes and allelic frequencies are given in Table S2. Additional information, including ethical authorizations for evolutionary and anthropological studies, can be found in the primary publication^58^.

### Population Branch Statistic (PBS) analysis

PBS determinations were performed between pair of populations, using Andean and Amazonian populations as sister groups and Mesoamericans as an outgroup. The analysis was carried out as described by Yi *et al*.^59^, with only the polymorphic SNPs in at least two of the populations being considered. From the genetic distances (*F*_*ST*_) between the three population groups examined, PBS measures if there are alleles with extreme frequencies in the Andean group as compared to the other two. Under a scenario of genetic drift only, we expect that Andeans and Amazonians will be more similar genetically than both compared to Mesoamericans. If, however, there has been local adaptation, we should detect genes that have been targeted by selection in Andeans. PBS values were estimated for both individual SNPs and windows of 20 SNPs overlapped in five SNPs. The empiric distribution of PBS values, with a 99.5^th^ threshold, was used to determine signals of positive selection (more details in Amorim *et al*.^60^).

### Demographic simulations

To verify the significance of the observed positive selection signals we simulated different demographic models, according to reported historical population data and inferred effective population sizes. We adapted the models described by Valverde *et al*.^11^, to account for the divergence between Mesoamericans, Andeans and Amazonians. Assuming that the American continent was peopled beginning at 15,000 yBP, the Andes colonized by 12,000 yBP and the Amazon by 10,000 yBP, and based on Nes estimated by Valverde *et al*.^11^, we simulated the three demographic models proposed by them: (a) Constant Model: Ne of 7,000 individuals with constant size in all populations throughout history; (b) Bottleneck Model: Ne 8,000 in Mesoamerica, 4,000 in Andes and 2,000 in Amazon; and (c) Bottleneck + Expansion Model: model b with bottlenecks reducing the effective size of all populations by 50% in the last 10,000 years followed by a sharp expansion in the last 8,000 years. Simulations were performed in the MS program^61^ with 10,000 replicates for each demographic scenario.

### Linkage disequilibrium analysis

We also used three linkage disequilibrium-based methods: extended haplotype homozygosity (EHH)^62^, integrated haplotype score (iHS)^63^, and cross-population extended haplotype homozygosity (XP-EHH)^64^. These approaches adopt the same core principle, that an advantageous allele under a hard sweep rise in frequency ─ carrying its neighbor alleles and therefore promoting homozygosity extension ─ quickly enough that recombination is not able to break down the haplotype. EHH statistics calculate the homozygosity rate from a core region (putative allele under selection) to the neutral scenery*, i.e*. the probability that any two randomly chosen chromosomes will be identical by descent, from the core region to a distance x. iHS evaluates the EHH considering both ancestral and derived alleles, and XP-EHH is used to calculate EHH/iHS between populations, therefore controlling for local variation. These tests are complementary; while iHS is better for detecting incomplete sweeps, XP-EHH has more power to detect sweeps near fixation^65^. Both measurements and significance were calculated through the ‘rehh’ R package^66^.

### Geographical analysis

To evaluate the variants spatial distribution, weighted inverse distance interpolation (IDW) was used to determine cell values using a weighted linear combination of a set of sample points. Weight is a function of the inverse distance^67^. The maps were made with the ArcGis 10.5 software and the cartographic base was georeferenced to the World Geodetic System (WGS84).

### Bootstrap Simulations

To verify whether the allele frequencies of the candidate variants under selection are significantly different among extreme high (>4,000 m) and lowland (<4,000 m) populations, we obtained the 95% confidence intervals of the average allele frequency of the lowland populations by means of 10,000 computer-assisted bootstrap simulations with replacement, considering a sample as having the same size and genotypic proportions observed in the real one. The average allele frequencies from high and lowland populations were obtained by weighing the observed frequencies according to their sample sizes.

### Analysis of gene expression

We used the Genotype-Tissue Expression Portal (GTEx; https://www.gtexportal.org/home/) to evaluate possible associations between each of the candidate alleles with highest differentiation and gene expression across human tissues looking for evidence of quantitative trait loci (eQTLs). The m-value is the posterior probability that an eQTL effect exists in each tissue tested in the cross-tissue meta-analysis. The m-value ranges between 0 and 1 (m-values > 0.9 mean that the tissue is predicted to have an eQTL effect).

## Electronic supplementary material

Supplementary Material
