# Supplementary material for: Selection scan reveals three new loci related to high altitude adaptation in Native Andeans
Source: Sci Rep. 2018 Aug 24;8:12733. doi: 10.1038/s41598-018-31100-6 (PMC6109162; doi:10.1038/s41598-018-31100-6)
Supplement: Supplementary file 1 — Supplementary Material [file 41598_2018_31100_MOESM1_ESM.pdf]

Supplementary Material

Selection scan reveals three new loci related to high altitude adaptation in Native Andeans

Vanessa C. Jacovas[1†], Cainã M. Couto-Silva[2†], Kelly Nunes[2†], Renan B. Lemes[2], Marcelo Z. de Oliveira[3], Francisco M. Salzano[1], Maria Cátira Bortolini[1*] & Tábita Hünemeier[2*]

[1]Genetics Departament, Biosciences Institute, Federal University of Rio Grande do Sul, Porto Alegre, RS, Brazil.

[2]Department of Genetics and Evolutionary Biology, Biosciences Institute, University of São Paulo, São Paulo, SP, Brazil.

[3]Vale do Rio dos Sinos University, São Leopoldo, RS, Brazil.

[†]These authors contributed equally to this work.

*Correspondence and requests for materials should be addressed to M.C.B. (email: maria.bortolini@ufrgs.br) or T.H. (email: hunemeier@usp.br)

Table S1. Significance of the PBS values for the extreme SNPs of each candidate gene, obtained for each simulated demographic model.

SNP (Gene)	Constant Model	Bottleneck+Expansion	Bottleneck
rs13411586 (*SP100*)	0.0001	0.0143	0.0074
rs10978240 (*TMEM38B*)	0.0017	0.0248	0.0147
rs1046778 (*AS3MT*)	0.0047	0.0315	0.0192
rs269866 (*DUOX2*)	0.0001	0.0132	0.0060
rs440191 (*CLC*)	0.0056	0.0321	0.0206

Table S2. Allelic frequencies by Native American population analyzed in the present study (*Weighted average).

**Population (n)**	***DUOX2* G allele (rs269866)**	***SP100* C allele (rs13411586)**	***CLC* G allele (rs440191)**
**Mesoamerican Lowland (< 2,500 m.)**			
Kaqchikel (13)	0	0.042	0
Maya (49)	0	0	0.138
Mixe (17)	0.059	0.029	0.147
Mixtec (5)	0.100	0.100	0.100
Purepecha (1)	0	0	0
Tepehuano (25)	0.240	0.020	0.080
Zapotec (43)	0.068	0.114	0.182
**Total (153)**	**0.068***	**0.045***	**0.128***
**South American (Andean) Highland (≥ 4,000 m.)**			
Aymara (23)	0.457	0.413	0.500
Quechua (40)	0.400	0.388	0.425
**Total (63)**	**0.420***	**0.397***	**0.452***
**South American (Amazonian) Lowland (< 2,500 m.)**			
Guahibo (6)	0	0	0.250
Guarani (6)	0.083	0	0.167
Jamamadi (1)	0	0.500	1
Kaingang (2)	0.500	0	0
Karitiana (13)	0	0.115	0
Kogi (4)	0	0	0.375
Maleku (3)	0	0	0
Palikur (3)	0	0.167	0.500
Parakana (1)	0	0.500	0
Piapoco (7)	0	0	0.286
Surui (24)	0	0	0
Teribe (3)	0	0.333	0
Ticuna (6)	0.167	0	0.167
Toba (4)	0.125	0.125	0.250
Waunana (3)	0	0.167	0.500
Wayuu (11)	0.056	0	0.056
Wichi (5)	0.200	0	0.300
Yaghan (4)	0.125	0.125	0.250
**Total (106)**	**0.048***	**0.053***	**0.142***

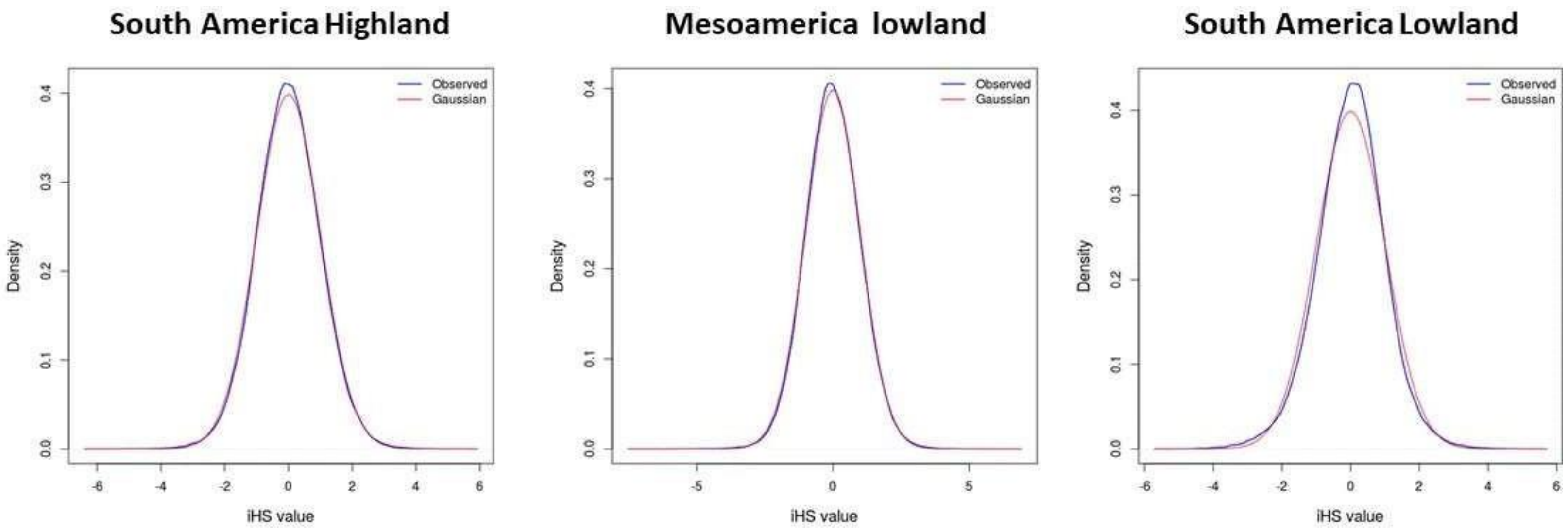

Figure S1. iHS value distribution patterns for all three groups (South American highland, South America Lowland and Mesoamerica Lowland).

	Mean (lowland)	Median (lowland)	95% c.i.	Mean (highland)
SP100 (rs13411586 C)	0.0656	0.0652	(0.0481; 0.0854)	0.3968
SP100 (rs9678342 C)	0.0637	0.0637	(0.0466; 0.0838)	0.3809
SP100 (rs7582700 T)	0.0965	0.0963	(0.0745; 0.1196)	0.3889
DUOX2 (rs269866 G)	0.0714	0.0714	(0.0528; 0.0916)	0.4206
CLC (rs440191 A)	0.1332	0.1335	(0.1071; 0.1599)	0.4524

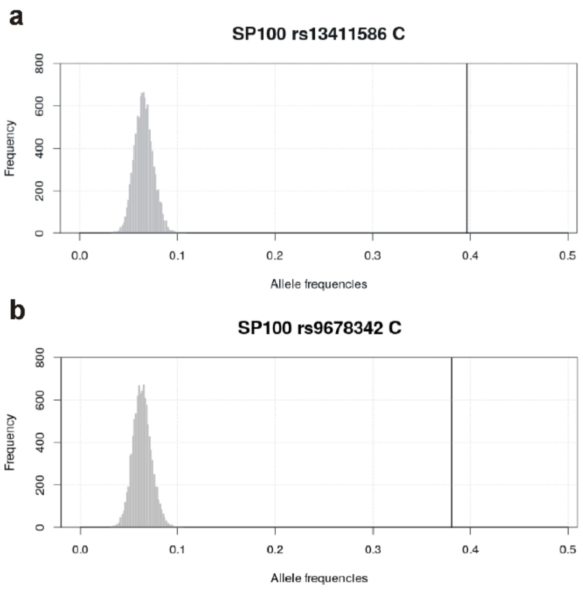

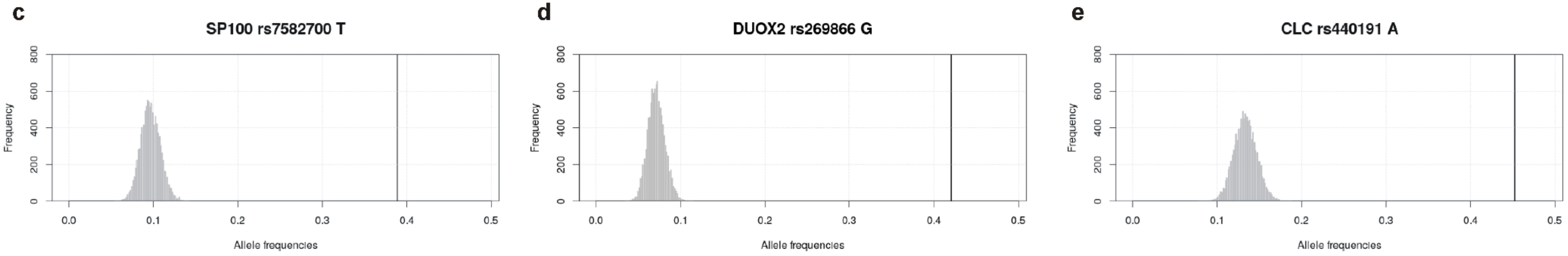

Figure S2. **Bootstrap simulations.** (A) Table showing average values (mean and median) of the allele frequencies for each SNP in lowland populations, as well as, their 95% confidence intervals obtained by simulation, and the average allele frequency of the candidate variant in highland populations. (B–F). Distribution of allele frequencies obtained by 10,000 simulations for lowlanders considering all markers in putative selection. The corresponding average allele frequencies observed for highland populations are represented by black vertical lines.

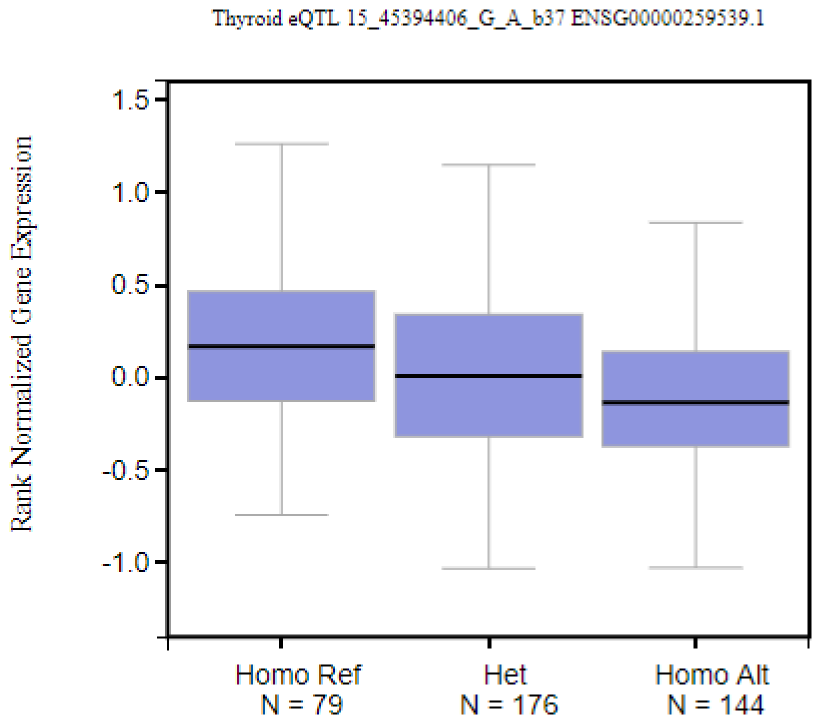

Figure S3. Differential expression of the DUOX2 putatively selected allele (rs269866 G) for a differential effect in gene expression in the thyroid tissue (https://www.gtexportal.org/home/, accessed 26/03/2018).

Tissue	Samples	NES	p-value	post-prob
Colon - Transverse	246	-	0.1	0.271
Colon - Sigmoid	203	-	0.4	0.413
Testis	225	-	0.3	0.311
Small Intestine - Terminal Ileum	122	-	0.8	0.693
Whole Blood	369	-	0.7	0.410
Muscle - Skeletal	491	-	0.8	0.431
Esophagus - Gastroesophageal Junction	213	-	0.9	0.596
Brain - Anterior cingulate cortex (BA24)	109	-	1	0.759
Stomach	237	-	0.7	0.340
Cells - Transformed fibroblasts	300	-	0.7	0.642
Pancreas	220	-	0.8	0.780
Heart - Left Ventricle	272	-	0.7	0.680
Brain - Putamen (basal ganglia)	111	-	0.7	0.848
Nerve - Tibial	361	-	0.4	0.759
Breast - Mammary Tissue	251	-	0.4	0.756
Adrenal Gland	175	-	0.6	0.792
Artery - Tibial	388	-	0.4	0.816
Spleen	146	-	0.6	0.795
Artery - Coronary	152	-	0.5	0.817
Esophagus - Mucosa	358	-	0.06	0.824
Brain - Spinal cord (cervical c-1)	83	-	0.6	0.786
Vagina	106	-	0.4	0.819
Brain - Substantia nigra	80	-	0.7	0.822
Brain - Frontal Cortex (BA9)	118	-	0.6	0.820
Minor Salivary Gland	85	-	0.3	0.815
Esophagus - Muscularis	335	-	0.1	0.870
Skin - Not Sun Exposed (Suprapubic)	335	-	0.01	0.992
Lung	383	-	1.1e-4	0.996
Heart - Atrial Appendage	264	-	0.1	0.912
Prostate	132	-	0.1	0.954
Skin - Sun Exposed (Lower leg)	414	-	1.7e-3	0.999
Adipose - Subcutaneous	385	-	0.06	0.973
Liver	153	-	0.3	0.933
Adipose - Visceral (Omentum)	313	-	0.04	0.961
Pituitary	157	-	0.2	0.922
Cells - EBV-transformed lymphocytes	117	-	0.2	0.926
Brain - Amygdala	88	-	0.3	0.889
Brain - Cortex	136	-	0.1	0.937
Thyroid	399	-	2.0e-7	1.00
Brain - Cerebellum	154	-	0.01	0.963
Ovary	122	-	0.07	0.942
Uterus	101	-	0.04	0.949
Brain - Hippocampus	111	-	0.02	0.952
Brain - Caudate (basal ganglia)	144	-	0.02	0.962
Artery - Aorta	267	-	1.7e-4	0.996
Brain - Nucleus accumbens (basal ganglia)	130	-	0.02	0.978
Brain - Cerebellar Hemisphere	125	-	1.1e-3	0.998
Brain - Hypothalamus	108	-	0.02	0.968

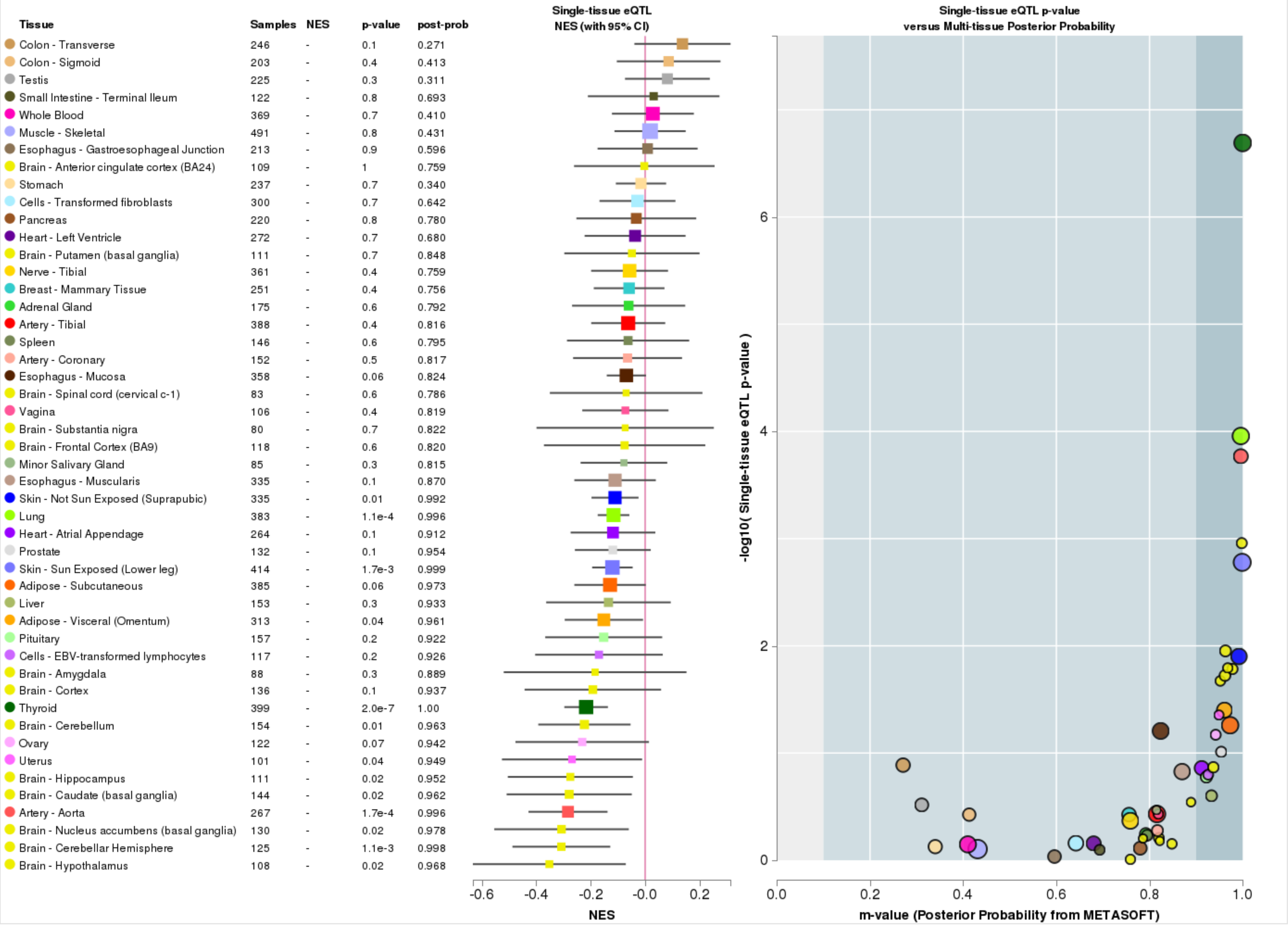

Figure S4. Posterior probabilities of DUOX2 putatively selected allele (rs269866 G) for a differential effect in gene expression in multiple tissues (https://www.gtexportal.org/home/, accessed 26/03/2018).

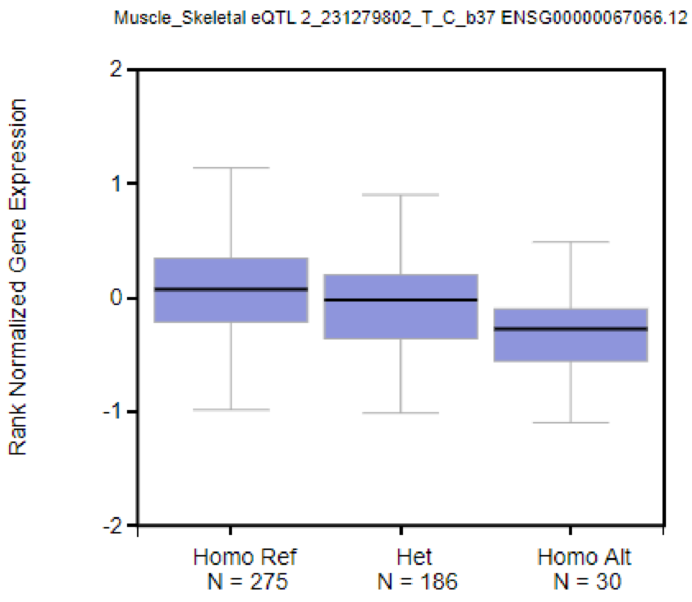

Figure S5. Differential expression of the SP100 putatively selected allele (rs13411586C) in the skeletal muscle (https://www.gtexportal.org/home/, accessed 26/03/2018).

Tissue	Samples	NES	p-value	post-prob
Artery - Coronary	152	-	3.4e-3	0.00
Cells - EBV-transformed lymphocytes	117	-	0.2	0.0270
Brain - Caudate (basal ganglia)	144	-	0.02	0.00
Brain - Cortex	136	-	0.1	0.00
Esophagus - Mucosa	358	-	0.009	0.00
Heart - Left Ventricle	272	-	0.1	0.0150
Brain - Putamen (basal ganglia)	111	-	0.1	0.00
Minor Salivary Gland	85	-	0.3	0.00
Liver	153	-	0.3	0.00
Lung	383	-	2.5e-3	0.00
Artery - Aorta	267	-	0.1	0.00
Adrenal Gland	175	-	0.3	0.00
Vagina	106	-	0.5	0.0100
Ovary	122	-	0.5	0.0220
Skin - Not Sun Exposed (Suprapubic)	335	-	0.3	0.00
Artery - Tibial	388	-	0.3	0.00
Stomach	237	-	0.6	0.00
Colon - Transverse	246	-	0.5	0.00
Small Intestine - Terminal Ileum	122	-	0.7	0.0240
Uterus	101	-	0.8	0.00600
Colon - Sigmoid	203	-	0.7	0.00
Whole Blood	369	-	0.5	0.00
Skin - Sun Exposed (Lower leg)	414	-	0.7	0.00
Brain - Substantia nigra	80	-	0.9	0.00400
Brain - Anterior cingulate cortex (BA24)	109	-	0.9	0.00600
Thyroid	399	-	0.9	0.00
Brain - Hippocampus	111	-	0.9	0.00
Brain - Amygdala	88	-	1	0.00400
Brain - Cerebellar Hemisphere	125	-	0.9	0.0200
Brain - Frontal Cortex (BA9)	118	-	0.9	0.0200
Heart - Atrial Appendage	264	-	0.9	0.00300
Spleen	146	-	0.9	0.105
Cells - Transformed fibroblasts	300	-	0.7	0.00100
Adipose - Subcutaneous	385	-	0.6	0.00100
Adipose - Visceral (Omentum)	313	-	0.5	0.00
Breast - Mammary Tissue	251	-	0.5	0.00
Brain - Nucleus accumbens (basal ganglia)	130	-	0.5	0.00900
Brain - Hypothalamus	108	-	0.5	0.0500
Brain - Cerebellum	154	-	0.5	0.0340
Nerve - Tibial	361	-	0.2	0.0460
Pancreas	220	-	0.2	0.00900
Prostate	132	-	0.4	0.100
Esophagus - Muscularis	335	-	1.5e-3	0.946
Brain - Spinal cord (cervical c-1)	83	-	0.09	0.324
Testis	225	-	8.1e-4	0.971
Esophagus - Gastroesophageal Junction	213	-	3.0e-3	0.872
Muscle - Skeletal	491	-	1.6e-6	1.00
Pituitary	157	-	0.007	0.746

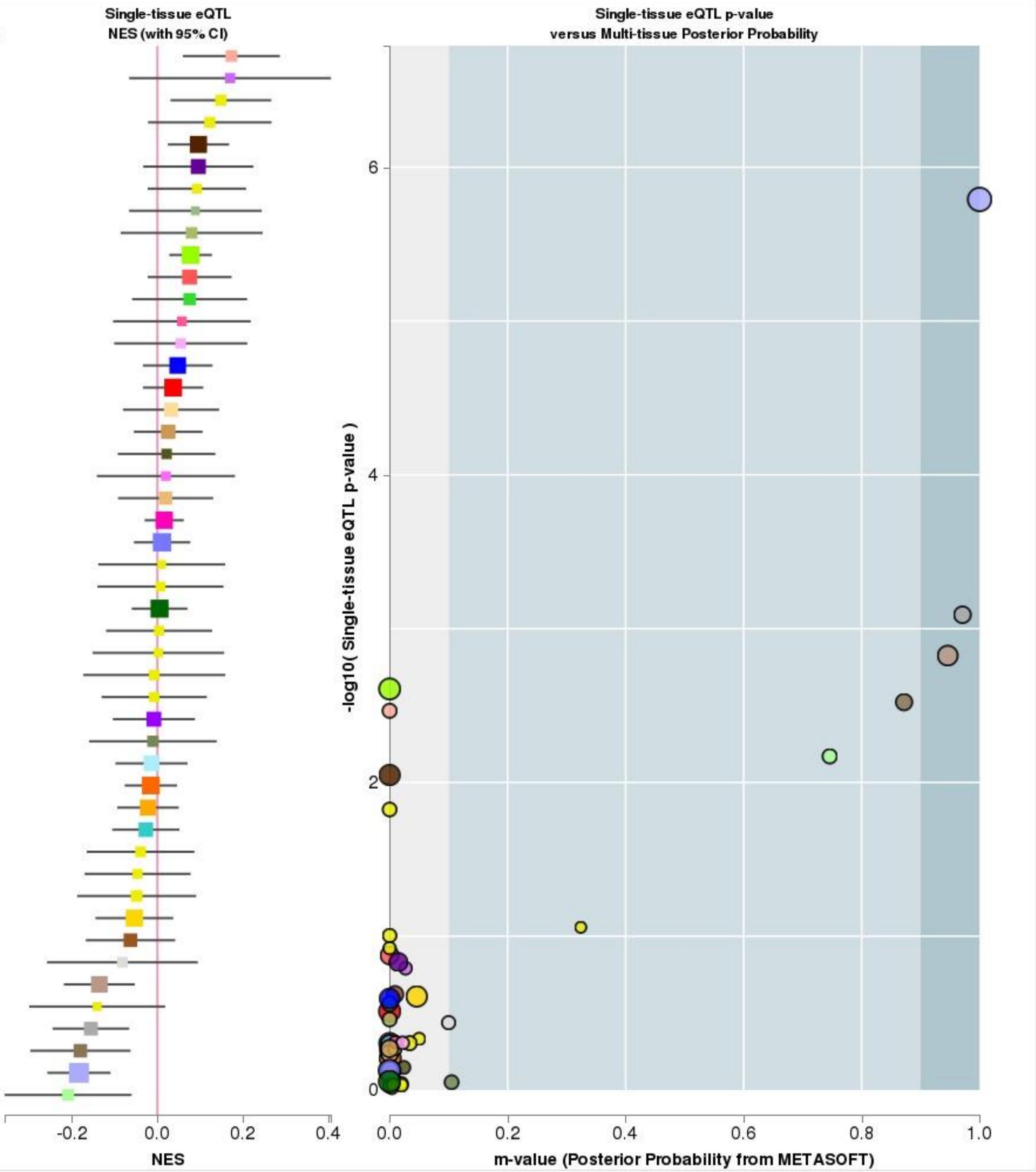

Figure S6. Posterior probabilities of SP100 putatively selected allele (rs13411586 C) for a differential effect in gene expression in multiple tissues (https://www.gtexportal.org/home/, accessed 26/03/2018).
